# Effect of Cochlear Implantation on Air Conduction and Bone Conduction Elicited Vestibular Evoked Myogenic Potentials—A Scoping Review

**DOI:** 10.3390/jcm13226996

**Published:** 2024-11-20

**Authors:** Muhammed Ayas, Jameel Muzaffar, Veronica Phillips, Mathew E. Smith, Daniele Borsetto, Manohar L. Bance

**Affiliations:** 1 College of Health Sciences, University of Sharjah, Sharjah P.O. Box 27272, United Arab Emirates; 2 Department of Clinical Neurosciences, University of Cambridge, Cambridge CB2 1TN, UK; 3 Cambridge Hearing Group, University of Cambridge, Cambridge CB2 1TN, UK; 4 Department of ENT, University Hospitals Birmingham NHS Foundation Trust, Birmingham B15 2TT, UK; 5 Department of Applied Health Sciences, University of Birmingham, Birmingham B15 2TT, UK; 6 Medical Library, School of Clinical Medicine, University of Cambridge, Cambridge CB2 0SP, UK; 7 Department of ENT, Cambridge University Hospitals NHS Foundation Trust, Cambridge CB2 0QQ, UK

**Keywords:** cochlear implants, vestibular function, bone conduction, vestibular evoked myogenic potential, VEMP, dizziness, electrical stimulation

## Abstract

**Background/Objectives:** Cochlear implantation (CI) is an effective intervention for individuals with severe to profound hearing loss; however, it may impact vestibular function due to its proximity to related anatomical structures. Vestibular evoked myogenic potentials (VEMPs) assess the function of the saccule and utricle, critical components of the vestibular system. This review examines CI’s impact on air conduction (AC) and bone conduction (BC) VEMP responses. **Methods:** A scoping review was conducted following PRISMA guidelines, using databases such as Medline, Embase, Cochrane Library, Scopus, and ProQuest Dissertations. Studies reporting on AC and/or BC-VEMP in CI recipients were included. Data extraction focused on VEMP response rates, amplitudes, and latencies pre- and post-CI. Risk of bias/quality assessment was performed using the Newcastle–Ottawa Scale. **Results:** Out of 961 studies identified, 4 met the inclusion criteria, encompassing a total of 245 CI-implanted ears. Results indicated that AC-VEMP responses were often reduced or absent post-CI, reflecting the influence of surgical changes in the middle ear mechanics rather than otolith dysfunction. In contrast, BC-VEMP responses were more consistently preserved, suggesting that BC stimuli bypass the middle ear and more accurately delineate otolith function. Variations in VEMP outcomes were noted depending on the surgical approach and individual patient factors. **Conclusions:** CI impacts vestibular function as measured by VEMP, with AC-VEMP showing greater susceptibility to postoperative changes compared to BC-VEMP. The presence of preserved BC-VEMP alongside absent AC-VEMP underscores the need to differentiate between these measures in assessing vestibular function.

## 1. Introduction

Cochlear implantation (CI) is a well-established intervention for individuals with severe to profound sensorineural hearing loss [1]. While the primary goal of CIs is to restore hearing, it is also important to consider the potential impact on the vestibular system. This concern arises from the close anatomical interconnection between the cochlea and the vestibular structures, with shared fluid spaces, which may lead to alterations in vestibular function following CI surgery [2]. Consequently, surgical interventions in the cochlea can inadvertently affect vestibular structures, namely the semicircular canals and the otolith organs (saccule and utricle) [3].

Surgical insertion of the electrode array can cause direct physical disruption to the delicate structures within the inner ear, which can also set up inflammatory processes. This trauma can also lead to changes in inner ear fluid hydrodynamics or changes in ionic environment. Some recipients report vestibular symptoms post-CI, such as dizziness and balance disturbances, while others experience no change or even improvements in their balance function [4,5]. Electrical stimulation of the auditory nerve may also stimulate the nearby vestibular nerve or hair cells. This cross-stimulation could also potentially modify the excitability of vestibular pathways to physiologic stimuli. The precise impact of this electrical stimulation varies among individuals, influenced by factors including electrode positioning and individual anatomical differences [6].

Vestibular evoked myogenic potentials (VEMPs) are used to assess the integrity and functionality of the utricular and saccular systems. VEMPs are categorized into two types: cervical VEMPs (VEMP), which primarily assess saccular function and the inferior vestibular nerve, and ocular VEMPs (VEMP), which primarily assess utricular function and the superior vestibular nerve [7]. These myogenic potentials can be elicited by both air conduction (AC) and bone conduction (BC) stimuli, providing alternative routes of stimulation to elicit responses from the vestibular system in individuals with or without CIs. Hereafter, VEMPs will be referred to as AC-VEMPs and BC-VEMPs throughout the text, while the terms “ocular” or “cervical” will be used where appropriate to specify which muscle groups the output was measured at.

Several studies have reported changes in AC-VEMPs following CI. Some patients exhibit reduced cervical AC-VEMPs amplitudes or absent responses post-CI, suggesting a possible impact on saccular function (thought to be best reflected in cervical VEMPs) due to surgical trauma or altered fluid dynamics within the inner ear [8,9,10]. Ocular AC-VEMPs may also show changes, and these are thought to reflect utricular involvement. These changes can be transient or persistent, indicating the need for long-term follow-up [11,12].

BC-VEMPs offers an alternative means of assessing vestibular function, particularly useful in cases where middle ear pathology might obscure AC-VEMPs results [13]. As BC stimulation bypasses the middle ear and directly stimulates the inner ear, BC-VEMPs are often more effective in detecting vestibular changes post-CI, as they stimulate the vestibular apparatus more directly. Studies have shown that BC-VEMP responses can also be affected by CI, with some patients exhibiting altered cervical BC-VEMP and ocular BC-VEMP amplitudes or latencies [13,14]. These findings underscore the complex interaction between CI and vestibular function, mediated by both mechanical and electrical factors.

These varying outcomes highlight the importance of individualized assessment and monitoring pre- and post-CI. The use of VEMPs to evaluate the vestibular impact of CI provides a noninvasive and objective measure of aspects of vestibular function. By comparing pre- and post-CI VEMP responses, we can gain valuable insights into the effects of CI surgery and subsequent electrical stimulation on vestibular structures [15]. Electrical stimulation can directly elicit VEMPs, thereby elucidating the spread of current to vestibular structures. Electrically evoked VEMPs can be elicited even when acoustic stimulation is unable to do so [15].

This scoping review aims to comprehensively examine and compare the effects of CI on AC and BC-VEMPs. We hypothesize that the observed absence of AC-VEMP responses post-CI can be attributed to alterations in peripheral auditory mechanics rather than a loss of vestibular function per se. Further, we explore the routine use of BC-VEMP in CI recipients for assessment of vestibular function.

## 2. Materials and Methods

### 2.1. Study Design

We conducted a scoping review based on the guidelines outlined in the preferred reporting items for systematic reviews and meta-analyses extension for scoping reviews (PRISMA-ScR) [16]. The review was prospectively registered in the PROSPERO database prior to the commencement of the literature search on 7 July 2023. The full protocol can be accessed via https://www.crd.york.ac.uk/prospero/display_record.php?RecordID=442978 (30 July 2024).

### 2.2. Study Inclusion Criteria

The literature search was carried out by outlining the PICO:

Population: Children and adult CI recipients;
Intervention: AC or/and BC-VEMP (cervical or ocular);
Comparison: AC/BC-VEMP amplitudes and latencies with before and after CI (Cervical-P13, N23 and Ocular-N10, and P16 latencies);
Outcome(s): Presence or absence of amplitudes and latencies AC/BC-VEMP before and after CI.

No restrictions were placed on study design for inclusion. Studies carried out as cross-sectional studies, longitudinal, experimental, quasi-experimental, and observational studies were all included in the review. Case reports, editorials, and studies not reported in English or lacking an English translation were excluded.

### 2.3. Search Strategy

The databases Medline (via Ovid), Embase (via Ovid), Cochrane Library, Scopus, and ProQuest Dissertations & Theses (via Web of Science) were searched from inception to August 2024 by VP. The search strategy was peer-reviewed by two librarian colleagues of VP using the Peer Review of Electronic Search Strategies (PRESS) checklist [17] and evaluated against the PRISMA-ScR guidelines (Supplementary Materials File S1). Databases were searched by VP separately, rather than multiple databases being searched on the same platform. The search syntax was adapted for each database and to account for variation between thesaurus terms/controlled vocabulary across each database. Results were imported to Endnote 21 by VP for deduplication, using the method outlined by Bramer et al. [18]. The searches were all rerun prior to submission in order to include any papers published between the initial searching and submission for peer review. The full search strategies used in each database are presented in the Supplementary Materials File S2. For example, the database search in Medline (via Ovid) was as follows.

1. ((cochlea* or auditory) adj5 (implant* or prosthe*)).ti,ab,kw—20,045;
2. Cochlear Implants/or Cochlear implantation/—16,663;
3. 1 or 2—21650;
4. (((“Air Conduct*” or AC or “bone conduct*” or BC or cervical or ocular) adj2 (“ves tibular evoked myogenic potential*” or VEMP)) or oVEMP or cVEMP).ti,ab,kw.—1382;
5. Vestibular Evoked Myogenic Potentials/or bone conduction/—5038;
6. 4 or 5—5486;
7. 3 and 6—300.

### 2.4. Study Selection

The outcomes of the database searches were transferred to the Rayyan web-based software (https://www.rayyan.ai) for independent and blinded eligibility screening. Duplicate studies were identified by Rayyan and checked manually prior to removal. Subsequently, title screening was performed independently by three authors (MA, JM, and DB). Abstracts of the included titles were screened and full-text articles were shortlisted and extracted. The reference lists of all full-text articles were further screened to identify any studies not highlighted by database searches. Discrepancies raised during the process of article screening were discussed and resolved through consensus by two authors (MS and MLB). Finally, articles which met the inclusion criteria were presented for full-text review and data extraction.

### 2.5. Data Extraction

The findings were recorded on a data collection sheet, created specifically for this study. The following information was extracted from the included studies: authors, year of publication, study location, sample size and characteristics, age of included participants, aetiology of hearing loss, CI surgical approach, cervical AC/BC-VEMP findings, ocular AC/BC-VEMP findings, and other vestibular tests used in the studies.

### 2.6. Quality Assessment

Studies were assessed for quality and risk of bias by the reviewers using the Newcastle–Ottawa Scale (NOS) [19]. The NOS assesses the quality of included studies in three areas: selection, comparability, and outcome. Additionally, the scale assesses the following: control cohort, the number of session (the length/follow up), and outcomes measures (objective or self-reported). The quality of the studies may be judged as either good (low risk), fair (high risk), or poor (very high risk) by awarding stars in each domain as per the NOS guidelines. No conflict of interest or financial incentives were declared in any of the primary studies. Discussion and consensus were reached between the authors when there was a discrepancy in the ratings assigned to studies.

## 3. Results

### 3.1. Sources of Evidence

A total of 961 records were identified through electronic database searches from various sources, including Medline (*n* = 300), Embase (*n* = 568), Cochrane (*n* = 6), Scopus (*n* = 81), and CINAHL (*n* = 6). After removing 291 duplicate records, 670 articles were screened based on their abstracts. Of these, 619 articles were excluded for not meeting the inclusion criteria. The primary reason for exclusion was the reporting of incomplete or insufficient VEMP data (*n* = 409). Specifically, these studies lacked essential details such as response rates, amplitudes, or latencies necessary for a comprehensive analysis of vestibular function. Additionally, 198 studies were excluded because they failed to evaluate key pre- and post-CI VEMP parameters relevant to assessing vestibular function in CI recipients and instead were reported as part of general vestibular assessment. Furthermore, 12 studies were excluded for examining the effects of CIs on vestibular function in pediatric populations only providing post CI VEMPs, as this fell outside the scope of this review.

**Figure 1:**
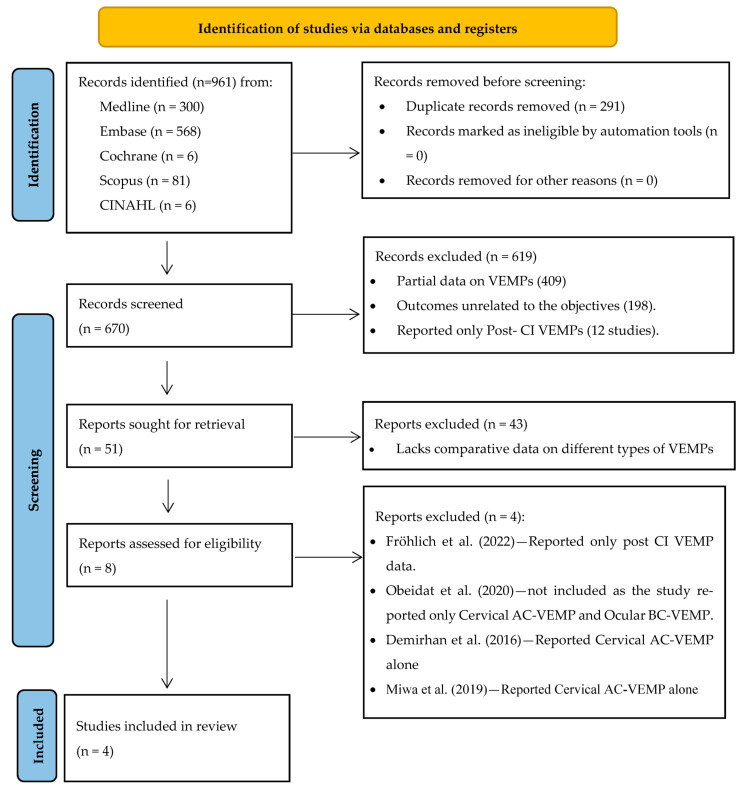
PRISMA flowchart for study identification and selection [20,21,22,23].

A total of 51 full-text records were sought for retrieval, of which 43 studies were excluded for lacking comparative data between different types of VEMPs. Eight full-text studies were assessed for eligibility and, during this evaluation, four studies were further excluded for reporting either AC-VEMP or BC-VEMP data alone without the necessary comparative context. Consequently, a total of four full-text articles met the inclusion criteria and were included in the final review. The screening, selection, and inclusion processes are illustrated in the PRISMA flowchart (Figure 1).

### 3.2. Characteristics of the Sources of Evidence

Table 1 summarizes the individual characteristics of the included studies. Studies were performed in Australia (1), Europe—Belgium (2), East Asia—Japan (3), and North America—United States (4). No studies were found from South America or from the Middle East and Africa. It is noteworthy that all published studies included in this review were conducted between 2020 and 2022. It is important to highlight that no date restrictions were applied to the initial search strategy, further highlighting current academic interest and the relevance of studies included in this review.

### 3.3. Synthesis of the Results

A total of 245 CI ears were reported across the four included studies, with an average patient age of 40.3 years. All studies employed a prospective study design. The hearing loss aetiologies included 38 cases of congenital hearing loss, 81 cases of acquired hearing loss, and 30 cases with unknown causes. The surgical approaches reported for these ears were primarily round window insertion (139 ears) and cochleostomy (24 ears), with two cases having unreported surgical methods. It is important to note that the count of 245 refers to individual ears (left or right) rather than individual patients, thus accounting for cases where bilateral CI may have been performed.

Vestibular function was assessed using a variety of tests. AC-VEMPs were measured pre- and post-CI in three studies. BC-VEMPs were tested in two studies pre- and post-CI. Additional vestibular tests included the dizziness handicap inventory (DHI), video head impulse test (vHIT), rotary chair, caloric testing, and wideband acoustic immittance (WAI).

### 3.4. AC and BC-VEMP

Reported VEMP response rates and amplitudes have varied significantly between participants with normal hearing and those that received CI. Participants with normal hearing consistently exhibited higher response rates for both cervical and ocular AC and BC-VEMPs compared to CI recipients, with statistical significance ranging from *p* = 0.036 to <0.001 [27]. Additionally, amplitude analysis showed that normal hearing participants had significantly higher amplitudes for both AC and BC-VEMPs (*p* < 0.001). Notably, BC-VEMP amplitudes in CI recipients were unaffected, whereas AC-VEMP amplitudes were significantly reduced.

### 3.5. Post-CI Amplitude and Latency Changes in VEMP

Post-CI changes in VEMP responses indicated that the majority of participants maintained their preoperative amplitude ratio (AR)—comparison of VEMP amplitudes between ears—across both AC and BC measurements. Specifically, 82.4% of participants retained their pre-CI AR for AC-VEMP, whereas 17.6% experienced a reduction of 30% or more post-CI (*p* = 0.029). In contrast, for BC-VEMP, 92.5% of participants maintained their pre-CI BC-VEMP levels, with only 7.5% experiencing a decrease post-CI (*p* = 0.64). This suggests that, while AC-VEMP responses showed a notable decrease in some participants, BC-VEMP responses remained relatively stable. However, it is important to note that this study did not provide data on pre- and post-CI BC-VEMP responses, which limits the ability to directly compare changes between AC and BC-VEMP outcomes.

Significant decreases in latency were observed in the ipsilateral latency of N1 in BC-VEMP (*p* = 0.027) [25], indicating a change post-CI. Additionally, significant decreases in AC-VEMP peak-to-peak amplitude were noted in 48% of CI recipients post-CI [24], with complete loss observed in 27% of participants [24]. Furthermore, 26% of participants had a complete loss of AC-VEMP responses [24]. It is important to note that a subset of participants with present BC-VEMP pre-CI demonstrated increased amplitude post-CI.

### 3.6. Surgical Approach on VEMP Responses

The two surgical approaches for electrode insertion reported in the included studies were the well-described round window and cochleostomy approaches. Three studies [24,25,26] predominantly used the round window technique, with well-preserved AC/BC-VEMP results observed post-CI in approximately 85% of the patients. In contrast, one study [27] used a cochleostomy approach in most cases and still reported present AC/BC-VEMP results in about 70% of patients. This suggests that, with a careful surgical technique, the cochleostomy approach can also help maintain vestibular integrity.

### 3.7. Quality of Studies

The quality assessment of four studies achieved good quality scores, demonstrating robust methodology across all domains, including cohort selection, exposure ascertainment, and follow-up adequacy. One study [26] scored slightly lower due to lesser comparability. The consistent high scores reflect strong study designs and reliable outcomes, ensuring a low risk of bias and high confidence in the findings presented. The quality assessment of all studies is summarized in Table 2.

## 4. Discussion

This systematic review explores the impact of CI on AC and BC-VEMP responses and compares the use of BC-VEMPs versus AC-VEMPs assessing vestibular integrity post-CI. This study further explores the ease of use of BC-VEMPs in routine practice and the influence of surgical approach on AC and BC VEMP responses.

### 4.1. Effect of CI on AC and BC VEMP

CI has been shown to influence VEMP responses, which are important measures for evaluating the integrity of the vestibular system post-CI. The four studies included in the review indicate that CI can negatively affect VEMP outcomes. The overall findings suggest that AC-VEMPs tend to decrease post-CI, while BC-VEMPs generally remain stable. This trend is supported by multiple studies reported in this review. For instance, one study [24] consistently reported reduced AR or complete loss of responses for AC-VEMPs both pre- and post-CI, whereas BC-VEMPs showed more stable outcomes. This suggests that CI might affect the ability to record AC-VEMPs, possibly due to the creation of an air–bone gap post-CI, as also observed by Merchant et al. [27], where AC-VEMP responses remained reduced in AR or as complete loss of responses post-CI.

While a small air–bone gap might be hard to measure with severe hearing losses, it might still affect acoustic energy flow to the vestibular structures or change the pressure distribution in the inner ear. Potential explanations for the air–bone gap include factors such as CI placement affecting compliance at the round window or altering how sound is transferred through the middle ear and cochlea, which might create an apparent air–bone gap [28,29]. Another possible explanation is that changes in compliance at the round window may be due to the placement of fascia [28]. While these changes in compliance may not have clinical relevance, since stimulation occurs electrically with the CI (except for electroacoustic stimulation), the resulting air–bone gap post-CI could impact AC-VEMP responses even when actual vestibular function remains normal, potentially leading to misdiagnosis. Additionally, although AC-VEMP responses were present pre- and post-CI in many cases, a significant reduction in AR was noted post-CI [26]. This further supports the idea that CI has a more pronounced impact on AC-VEMPs compared to BC-VEMPs.

In particular, the study by Dhondt et al. [25] emphasizes the importance of BC-VEMPs as a reliable measure of saccular integrity in children, both pre- and post-CI. This study suggests that BC-VEMPs can provide valuable clinical information, even when AC-VEMP results are compromised, likely due to factors such as middle ear effusion in children, which can influence AC-VEMP outcomes.

Interestingly, one study [25] reported that AC/BC-VEMP responses are better obtained post-CI when the device was turned off (“CI off” condition), avoiding interference from CI electrical stimulation during the recording. This finding may indicate hyperexcitability of the inferior vestibular nerve when the AC/BC-VEMPs are recorded when in the “CI on” condition [6]. Furthermore, some studies have suggested that the electrical stimulation from the CI device when recording AC/BC-VEMP might have crossover effects on contralateral AC/BC-VEMP responses [30,31], potentially affecting central vestibular processing [32], though this remains an area for further exploration. Consideration of these factors during clinical recording of AC and BC-VEMP responses may help in interpreting obtained AR and any potential shift in VEMP latency responses in CI recipients. These findings also highlight the importance of considering comprehensive pre-CI vestibular assessments to identify potential risks and tailor post-CI rehabilitation strategies accordingly.

### 4.2. Influence of Surgical Approach on VEMP and Vestibular Function

Potential risks associated with CI surgery to the vestibular system include direct trauma during electrode insertion, labyrinthitis due to foreign body reaction or biofilms [33], disturbances in inner ear fluids, or effects caused by electrical stimulation from the implant itself. Thus, the surgical approach to CI electrode insertion plays an important role in preserving vestibular function [34]. The studies included in this review present mixed results regarding the impact of the surgical approach on AC/BC-VEMP responses and overall vestibular function. Round window and cochleostomy approaches were used for electrode insertion. Three studies [24,25,26] with the round window technique reported well-preserved AC/BC-VEMP post-CI. In theory, this approach may be associated with less trauma to the vestibular system [34], potentially improving the chances of preserving vestibular function when compared to cochleostomy.

In contrast, one study [27] used a cochleostomy approach in most cases and still reported positive AC/BC-VEMP results, suggesting that, with a careful surgical technique, this approach can also maintain vestibular integrity. The variability in outcomes between the studies suggests that, whilst the surgical approach is an important factor, the skill and experience of the surgeon, along with the patient’s anatomy and pathology, plays a significant role in determining the impact on vestibular function [34,35]. It would be valuable if future studies should report more detailed surgical technique data, including hearing preservation techniques employed such as intratympanic steroid, electrode type (lateral vs. medial wall), and challenges encountered during insertion. Additionally, reporting on the immediate and long-term effects of these variables on vestibular function will help to establish clearer guidelines on CI surgical practice and improve patient outcomes.

### 4.3. Clinical Utility of BC-VEMP and AC-VEMP

The comparison between BC and AC-VEMPs for the assessment of CI recipients demonstrates more reliable responses for BC-VEMPs, suggesting its potential superior utility in the clinical setting. Results were more consistent with preserved BC-VEMP responses throughout, suggesting that AC-VEMPs might be more sensitive to individual differences in vestibular system integrity [24,25,27] or the specific details of the CI procedure [26]. The degree of robustness and reliability of BC measures suggest that BC-VEMP responses might be a more stable and less variable measure compared to AC-VEMPs in CI recipients [25].

Studies suggest that using BC stimulation instead of, or in addition to, AC stimulation to elicit VEMP responses would yield improved results and overcome the discrepancy in responses obtained using AC-VEMPs alone in CI recipients. Given that the saccule may potentially be more vulnerable to damage during CI surgery, as evidenced by loss of cervical AC/BC-VEMPs more than ocular AC/BC-VEMPs, assessing and monitoring saccular integrity is important for assessing holistic outcomes in CI [27,36].

However, caution is important here due to reported uncertainty regarding the origin of AC and BC responses in VEMPs. Some researchers suggest that AC stimulation would yield a more focussed triggering of saccular afferents, whereas BC stimulation may demonstrate potentially greater influence of saccular as well as utricular afferents in its stimulation process [37].

The superiority of one method over the other cannot be definitively concluded from currently available data. Each method has its own advantages, with AC-VEMPs being more commonly used and providing consistent results, while BC-VEMPs might offer additional insights in certain cases, particularly where the risk of misdiagnosis exists when relying solely on AC-VEMPs. A combined approach utilizing both AC and BC-VEMPs particularly in post-CI VEMP recording could potentially provide a more comprehensive assessment of vestibular function and better inform clinical decisions pre- and post-CI.

### 4.4. Clinical Implications

Understanding the effect of CI on AC and BC-VEMP has important clinical implications. For instance, pre-CI BC-VEMP testing can help to provide a baseline for patients who may be at higher risk of post-CI vestibular dysfunction. This would allow for better-informed patient counselling. Further, post-CI BC-VEMP monitoring can aid in the early detection of vestibular changes, guiding rehabilitation strategies to mitigate balance-related issues. Finally, incorporating BC-VEMPs into routine clinical practice would enhance the assessment of saccular function post-CI as a measure of the traumatic effect of implantation.

### 4.5. Limitations

Despite the insights gained from this scoping review, several limitations must be acknowledged. The review includes only four studies, limiting the generalizability of the findings. The heterogeneity in study designs, participant demographics, and VEMP testing protocols may impede direct comparisons and prevented meta-analysis of results. Variations in post-CI follow-up periods may overlook long-term effects on vestibular function. Further, the effect of CI ON vs. OFF VEMP recording protocol was not explored in all studies to measure the effect of CI stimulation on VEMP responses.

### 4.6. Future Directions

Future research should focus on longitudinal studies with larger sample sizes to better understand the long-term effects of CI on vestibular function using VEMPs. Additionally, exploring the impact of different CI electrode designs and surgical techniques on vestibular outcomes could help optimize patient care. Exploring the effect of CI ON vs. OFF VEMP recording protocols to measure the effect of CI stimulation on VEMP responses would yield a better understanding of vestibular physiological changes during electrical stimulation. CIs may also influence the inner ear via other mechanisms, such as inflammatory reactions, infections, or biofilms.

## 5. Conclusions

The effect of CI on AC- and BC-elicited VEMPs is complex and involves multiple contributing factors. While CIs provide significant auditory benefits, their impact on vestibular function necessitates careful consideration. AC-VEMP responses show greater susceptibility to postoperative changes compared to BC-VEMP responses. The presence of preserved BC-VEMPs alongside absent AC-VEMPs underscores the importance of differentiating these measures in assessing vestibular function. Comprehensive pre- and post-CI VEMP testing, particularly with BC stimulation, enables clinicians to better understand and manage vestibular issues, ultimately reducing the risk of balance disturbances.

## Figures and Tables

**Table 1:** Characteristics of the included studies.

Studies	Location	Sample (CI Ears)	Age (Mean)	Aetiology			Surgical Approach			Cervical AC-VEMP		Cervical BC-VEMP		Ocular AC-VEMP		Ocular BC-VEMP		Other Vestibular Tests	
				Congenital	Acquired	Unknown	Round window	Cochleostomy	Unknown	Pre CI	Post CI	Pre CI	Post CI	Pre CI	Post CI	Pre CI	Post CI	Pre CI	Post CI
Kwok et al. 2022 [24]	Australia	72	59	7	65	NR	71	1	0	Yes	Yes	Yes	Yes	Yes	Yes	Yes	Yes	DHI, vHIT	DHI, vHIT
Dhondt et al. 2022 [25]	Belgium	80	<17	20	16	14	NR	NR	0	No	No	Yes	Yes	No	No	No	No	Rotary Chair	Rotary Chair
Tsukada et al. 2021 [26]	Japan	66	46.6	NR	NR	NR	66	0	0	Yes	Yes	No	No	No	No	Yes	Yes	Caloric testing	Caloric testing
Merchant et al. 2020 [27]	USA	27	15.29	11	NR	16	2	23	2	Yes	Yes	Yes	Yes	Yes	Yes	Yes	Yes	WAI	WAI

NR—no response; AC—air conduction; BC—bone conduction; VEMP—vestibular myogenic potentials; CI—cochlear implants; DHI—dizziness handicap inventory; vHIT—video head impulse test; WAI—wideband acoustic immittance.

**Table 2:** Quality and risk of bias assessment (Newcastle–Ottawa Scale) criteria.

Author	Selection				Comparability	Outcome			Total Quality Score	
	Representativeness of the Exposed Cohort	Selection of the NonExposed Cohort	Ascertainment of Exposure	Demonstration that Outcome of Interest Was Not Present at Start of Study	Comparability of Cohorts on the Basis of the Design or analysis	Assessment of Outcome	Was Follow-up Long Enough for Outcomes to Occur	Adequacy of Follow up of Cohorts		
Kwok et al. 2022 [24]	*	*	*	*	*	*	*	*	8	Good
Dhondt et al. 2022 [25]	*	*	*	*	*	*	*	*	8	Good
Tsukada et al. 2021 [26]	*	-	*	*	*	*	*	*	7	Good
Merchant et al. 2020 [27]	*	*	*	*	*	*	*	*	8	Good

NOS has a total maximum score of 9: maximum scores 4 in Selection, 2 in Comparability, and 3 in Outcome. Studies scoring 7–9 have good quality (high quality), 4–6 have fair quality (high risk), and 0–3 have poor quality (very high risk). The symbol (*) means the point earned in each category and (-) no points.

## Data Availability

Data are contained within the article and Supplementary Materials.

## Supplementary Materials

The following supporting information can be downloaded at: https://www.mdpi.com/article/10.3390/jcm13226996/s1, Supplementary File S1: Prisma Scoping review list; Supplementary File S2: Search results.

## Abbreviations

AC	Air conduction
AR	Amplitude Ratio
BC	Bone Conduction
CI	Cochlear implants
VEMP	Vestibular Evoked Myogenic Potentials
