# Supplementary material for: Effect of Cochlear Implantation on Air Conduction and Bone Conduction Elicited Vestibular Evoked Myogenic Potentials—A Scoping Review
Source: J Clin Med. 2024 Nov 20;13(22):6996. doi: 10.3390/jcm13226996 (PMC11595251; doi:10.3390/jcm13226996)
Supplement: Supplementary file 1 [file jcm-13-06996-s001.zip › Supplementary File S2-Search results.pdf]

Supplementary material -2

Search Methods

Ovid MEDLINE(R) and Epub Ahead of Print, In-Process, In-Data-Review & Other Non-Indexed Citations, Daily and Versions <1946 to August 01, 2024>

1 ((cochlea* or auditory) adj5 (implant* or prosthe*)).ti,ab,kw. 20045
2 Cochlear Implants/ or Cochlear implantation/ 16663
3 1 or 2 21650
4 ((("Air Conduct*" or AC or "bone conduct*" or BC or cervical or ocular) adj2 ("vestibular evoked myogenic potential*" or VEMP)) or oVEMP or cVEMP).ti,ab,kw. 1382
5 Vestibular Evoked Myogenic Potentials/ or bone conduction/ 5038
6 4 or 5 5486
7 3 and 6 300

Embase <1974 to 2024 August 01>

1 ((cochlea* or auditory) adj5 (implant* or prosthe*)).ti,ab,kw. 23080
2 Cochlea prosthesis/ or Cochlear Implantation/ 22491
3 1 or 2 26558
4 ((("Air Conduct*" or AC or "bone conduct*" or BC or cervical or ocular) adj2 ("vestibular evoked myogenic potential*" or VEMP)) or oVEMP or cVEMP).ti,ab,kw. 1688
5 Vestibular Evoked Myogenic Potential/ or bone conduction/ or air conduction/ 10069
6 4 or 5 10210
7 3 and 6 568

Cochrane Library

((cochlea* or auditory) Near/5 (implant* or prosthe*)):ti,ab,kw OR MeSH descriptor: [Cochlear Implants] this term only or MeSH descriptor: [Cochlear Implantation] this term only
AND
(("Air Conduct*" OR AC OR "bone conduct*" OR BC OR cervical OR ocular) Near/2 ("vestibular evoked myogenic potential*" OR VEMP) OR oVEMP OR cVEMP):ti,ab,kw OR MeSH descriptor: [Vestibular Evoked Myogenic Potentials] this term only OR MeSH descriptor: [Bone Conduction] this term only

Scopus

(TITLE-ABS-KEY ((("Air Conduct*" OR ac OR "bone conduct*" OR bc OR cervical OR ocular) W/2 ("vestibular evoked myogenic potential*" OR vemp) OR ovemp OR cvemp))) AND (TITLE-ABS-KEY ((cochlea* OR auditory) W/5 (implant* OR prosthe*)))

# Web of Science Search Strategy (v0.1)

# Database: ProQuest ™ Dissertations & Theses Citation Index

# Entitlements:

- PQDT.PQDT: 1637 to 2024

# Searches:

1: TS=(((cochlea* or auditory) near/5 (implant* or prosthe*))) Date Run: Fri Aug 02 2024 09:12:07 GMT+0100 (British Summer Time) Results: 1230

2: TS=((("Air Conduct*" OR AC OR "bone conduct*" OR BC OR cervical OR ocular) Near/2 ("vestibular evoked myogenic potential*" OR VEMP) OR oVEMP OR cVEMP)) Date Run: Fri Aug 02 2024 09:12:24 GMT+0100 (British Summer Time) Results: 33

3: #2 AND #1 Date Run: Fri Aug 02 2024 09:12:33 GMT+0100 (British Summer Time) Results: 1
